# Supplementary material for: Preferential retention of genes from one parental genome after polyploidy illustrates the nature and scope of the genomic conflicts induced by hybridization
Source: PLoS Genet. 2018 Mar 28;14(3):e1007267. doi: 10.1371/journal.pgen.1007267 (PMC5891031; doi:10.1371/journal.pgen.1007267)
Supplement: S1 Fig — Shown is the assumed 11 species topology for yeast and the maximum likelihood topology for ρ, with branch lengths estimated as in Fig 1. Above each branch is the estimated number of genes returned to single copy with the gene from parental subgenome 1 being retained, while below each branch is the corresponding number for subgenome 2. These data were inferred from model WGD-btf (e.g., a model with fixation and biased fractionation where the biased fractionation rate differs on the root branch, red: εearly, compared to the remainder of the tree, blue: εlate) for yeast and WGD-bf for the grass ρ event (see Figs 1 & 2). (PDF) [file pgen.1007267.s001.pdf]

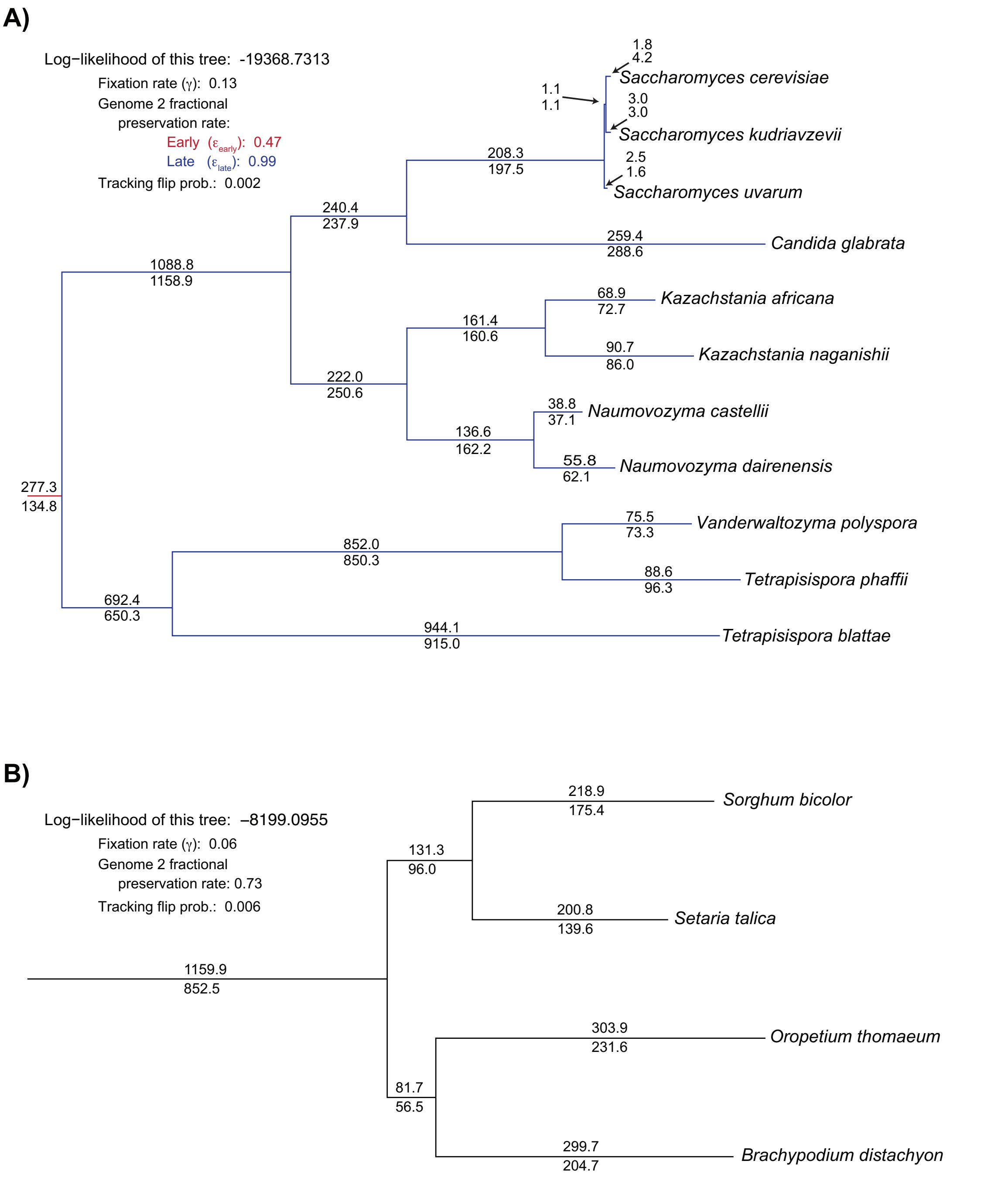

S1 Fig: Gene loss patterns after the yeast (**A**) and grass ρ (**B**) WGD events. Shown is the assumed 11 species topology for yeast and the maximum likelihood topology for ρ, with branch lengths estimated as in Figure 1. *Above* each branch is the estimated number of genes returned to single copy with the gene from parental subgenome 1 being retained, while *below* each branch is the corresponding number for subgenome 2. These data were inferred from model WGD-b_tf (e.g., a model with fixation and biased fractionation where the biased fractionation rate differs on the root branch, red: ε_{early}, compared to the remainder of the tree, blue: ε_{late}) for yeast and WGD-*bf* for the grass ρ event (see Figures 1&2).
