# Supplementary material for: Preferential retention of genes from one parental genome after polyploidy illustrates the nature and scope of the genomic conflicts induced by hybridization
Source: PLoS Genet. 2018 Mar 28;14(3):e1007267. doi: 10.1371/journal.pgen.1007267 (PMC5891031; doi:10.1371/journal.pgen.1007267)
Supplement: S2 Fig — All details are otherwise as for Fig 4 in the main text. Blocks for the both the WGD-bf (blue/green) and the WGD-f (pink/purple) models are shown for the At-α and ρ events. Because the global WGD-bf model showed little evidence for BF in yeast, we illustrate the inferred blocks from the WGD-btf model for these taxa: note the lack of subgenome resolution due to the balanced gene losses seen on most of the branches of S1 Fig. (PDF) [file pgen.1007267.s002.pdf]

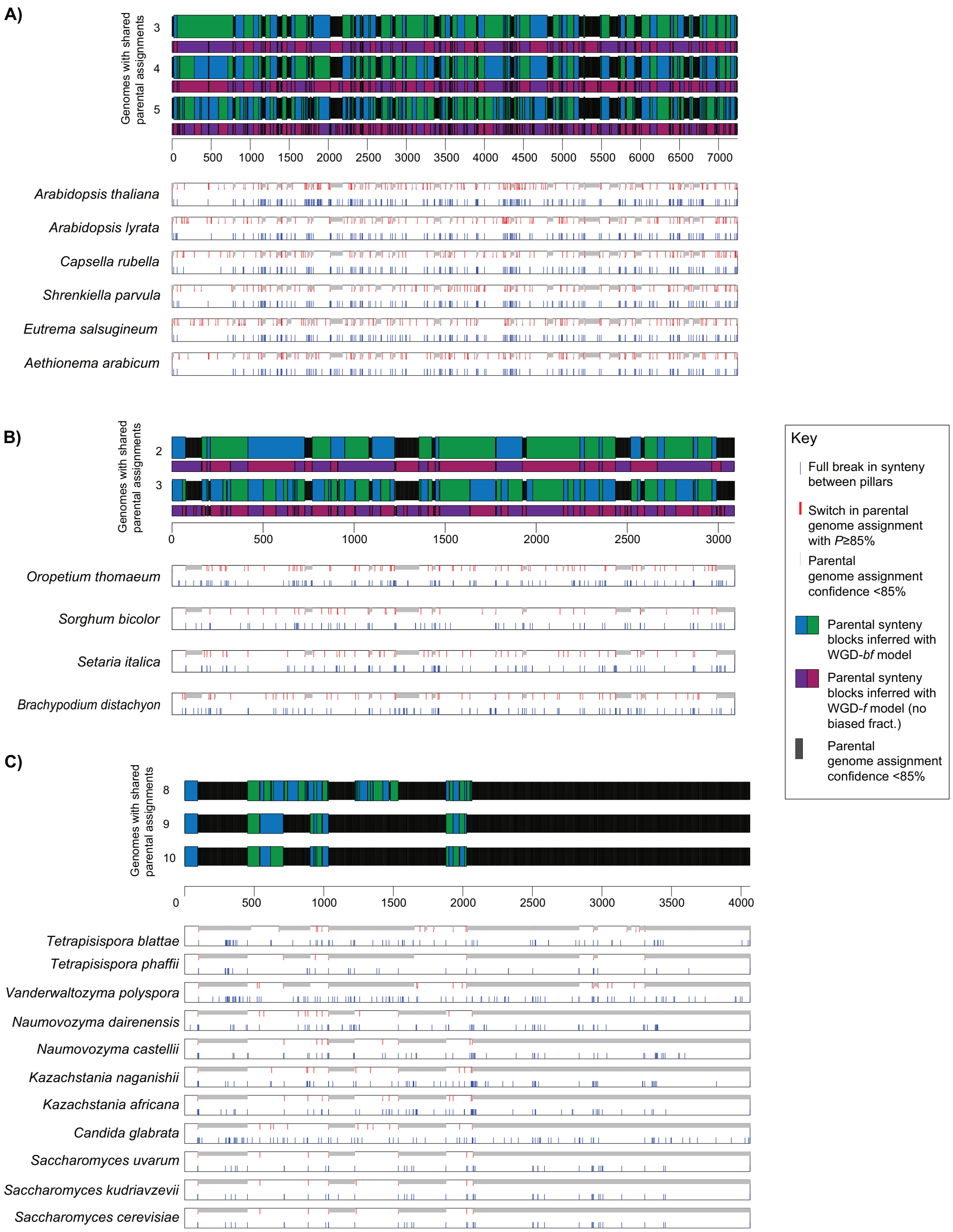

S2 Fig: Consistency of POInT's estimates of the parental genome of origin for each species individually (lower part) and for the combination of species (upper) for the full At-α dataset (panel **A**), the grass ρ event (panel **B**) and for the yeast WGD (panel **C**). All details are otherwise as for Figure 4 in the main text. Blocks for the both the WGD-*bf* (blue/green) and the WGD-*f* (pink/purple) models are shown for the At-α and ρ events. Because the global WGD-*bf* model showed little evidence for BF in yeast, we illustrate the inferred blocks from the WGD-$b_t f$ model for these taxa: note the lack of subgenome resolution due to the balanced gene losses seen on most of the branches of Supplemental Figure 1.
