# Supplementary material for: Preferential retention of genes from one parental genome after polyploidy illustrates the nature and scope of the genomic conflicts induced by hybridization
Source: PLoS Genet. 2018 Mar 28;14(3):e1007267. doi: 10.1371/journal.pgen.1007267 (PMC5891031; doi:10.1371/journal.pgen.1007267)
Supplement: S3 Fig — A) Molecular function hierarchy. B) Biological processes hierarchy. On the y axis is the ln(fold-enrichment) of the term in question among the single copy genes relative to the duplicates. Blue dots represent terms that are significantly over/underrepresented in single copy genes relative to duplicates with Bonferroni corrected P-value ≤ 0.01 and are >1.5 fold over/underrepresented. On the x-axis is the ln(fold-enrichment) of GO terms of genes from subgenome 1 relative to those from subgenome 2. The GO terms that are over/underrepresented in genes from subgenome 1 (the less fractionated subgenome) with P-value ≤ 0.05 after Bonferroni correction are shown in triangles. GO terms that are significantly different both between the single copy and duplicate genes and between subgenome 1 and subgenome 2 are shown in dark purple. (PDF) [file pgen.1007267.s003.pdf]

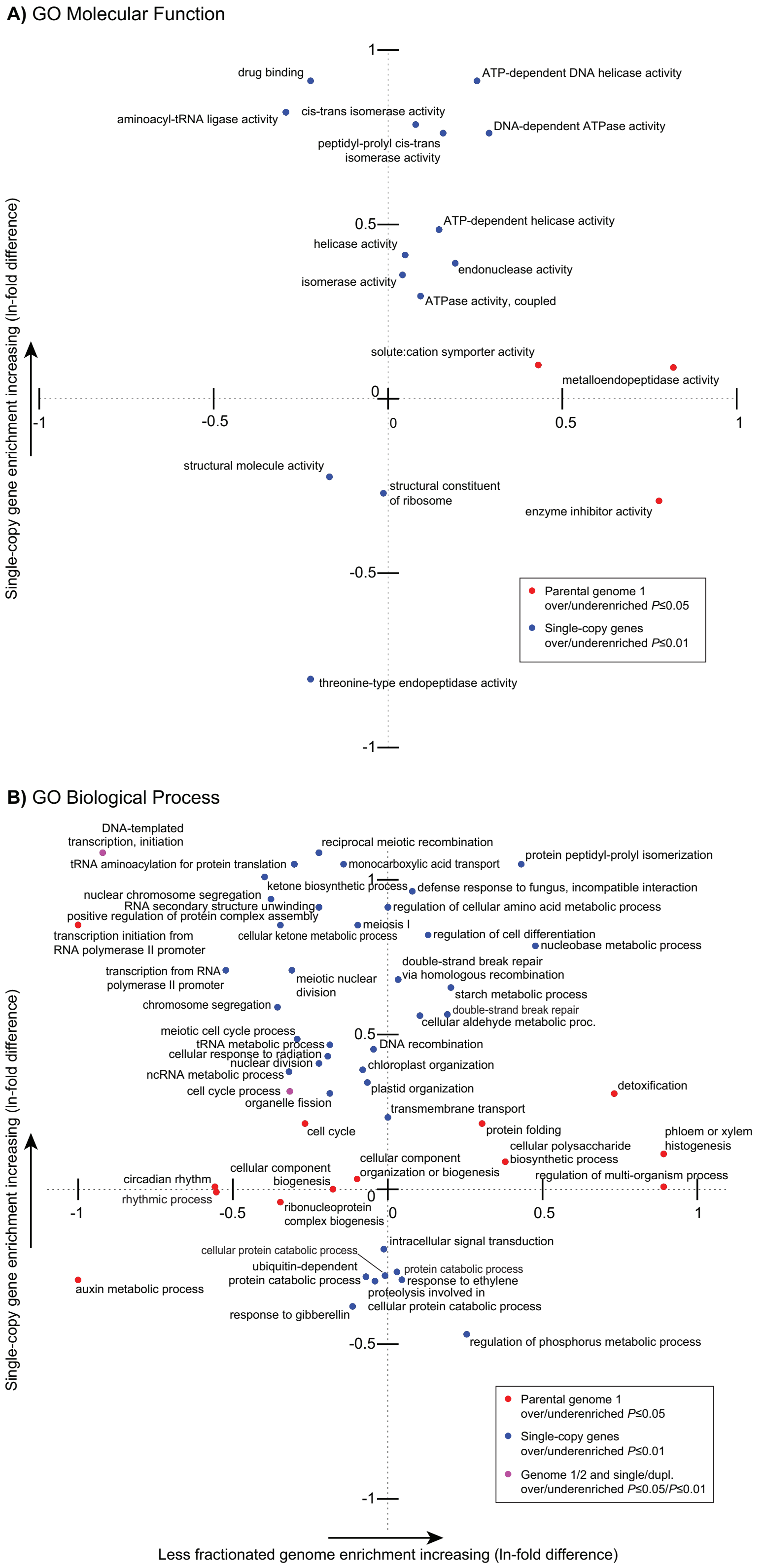

S3 Fig: Statistically overrepresented GO terms associated with At-α duplication status and parental genome of origin (see *Methods*) **A)** Molecular function hierarchy. **B)** Biological processes hierarchy. On the *y* axis is the ln(fold-enrichment) of the term in

question among the single copy genes relative to the duplicates. Blue dots represent terms that are significantly over/underrepresented in single copy genes relative to duplicates with Bonferroni corrected P-value ≤ 0.01 and are >1.5 fold over/underrepresented. On the *x*-axis is the ln(fold-enrichment) of GO terms of genes from subgenome 1 relative to those from subgenome 2. The GO terms that are over/underrepresented in genes from subgenome 1 (the less fractionated subgenome) with P-value ≤ 0.05 after Bonferroni correction are shown in triangles. GO terms that are significantly different both between the single copy and duplicate genes and between subgenome 1 and subgenome 2 are shown in dark purple.
