# Supplementary material for: Preferential retention of genes from one parental genome after polyploidy illustrates the nature and scope of the genomic conflicts induced by hybridization
Source: PLoS Genet. 2018 Mar 28;14(3):e1007267. doi: 10.1371/journal.pgen.1007267 (PMC5891031; doi:10.1371/journal.pgen.1007267)
Supplement: S1 Table — (DOCX) [file pgen.1007267.s004.docx]

**S1 Table:** Effect of inferred ancestral order on POInT estimates

| **Description** | **# breaks^a^** | **#double breaks^b^** | **WGD-*bf* lnL^c^** | **Fixation rate (γ)^d^** | **Bias strength (ε)^e^** |
| --- | --- | --- | --- | --- | --- |
| *C. violacea order* | 6614 | 3021 | -25357.46 | 0.160 | 0.538 |
| Opt_13_ | 5485 | 1179 | -24551.65 | 0.170 | 0.648 |
| **Opt_14_^f^** | **5468** | **1129** | **-24497.04** | **0.169** | **0.645** |
| Opt_15_ | 5446 | 1113 | -24553.34 | 0.170 | 0.662 |
| Opt_16_ | 5418 | 1169 | -24585.75 | 0.170 | 0.664 |
| Opt_17_ | 5382 | 1144 | -24551.59 | 0.170 | 0.663 |
| Opt_18_ | 5367 | 1147 | -24568.93 | 0.169 | 0.649 |
| Opt_19_ | 5345 | 1117 | -24553.55 | 0.170 | 0.667 |
| Opt_20_ | 5337 | 1098 | -24541.95 | 0.169 | 0.666 |
| Opt_21_ | 5294 | 1058 | -24517.59 | 0.170 | 0.662 |
| Opt_22_ | 5282 | 1060 | -24554.87 | 0.172 | 0.674 |
| Opt_23_ | 5269 | 1060 | -24520.32 | 0.170 | 0.663 |

a: Number of synteny breaks across the six genomes.

b: Number of cases where both parental genomes showed a synteny break after an ancestral locus.

c: log-likelihood from fitting WGD-*bf* to this ancestral order.

d: Maximum likelihood estimate of the relative duplicate fixation rate for this order (see Figure 1).

e: Maximum likelihood estimate of the relative rate of retention from the more fractionated genome for this order (see Figure 1).

f: This ancestral order had the maximum likelihood.
