# Supplementary material for: Preferential retention of genes from one parental genome after polyploidy illustrates the nature and scope of the genomic conflicts induced by hybridization
Source: PLoS Genet. 2018 Mar 28;14(3):e1007267. doi: 10.1371/journal.pgen.1007267 (PMC5891031; doi:10.1371/journal.pgen.1007267)
Supplement: S2 Table — (DOCX) [file pgen.1007267.s005.docx]

**S2 Table:** Modeling convergent losses with POInT

| **Dataset** | **WGD-*bf*^a^** | | | **WGD-*bfc^b^*** | | | | ***P^c^*** |
| --- | --- | --- | --- | --- | --- | --- | --- | --- |
|  | **lnL^d^** | **Fixation rate (γ)^e^** | **Bias strength (ε)^f^** | **lnL^d^** | **Fixation rate (γ)^e^** | **Bias strength (ε)^f^** | |  |
| At-α, Full | -24497.04 | 0.169 | 0.645 | -24171.37 | 0.200 | | 0.702 | <10^-10^ |
| At-α, High-syn | -12442.51 | 0.220 | 0.786 | -12332.05 | 0.159 | | 0.818 | <10^-10^ |
| Grass ρ | -8199.10 | 0.061 | 0.730 | -8099.91 | 0.079 | | 0.760 | <10^-10^ |
| Yeast | -19374.10 | 0.137 | 0.955 | -19040.17 | 0.057 | | 0.999 | <10^-10^ |

a: Values from the biased-fractionation and fixation model (Figure 1B).

b: Values from the biased-fractionation, convergence and fixation model. This model adds duplicated states C_1_ and C_2_ where losses from C_1_ are always to S_1_ and similarly for C_2_. This model adds two parameters relative to WGD-*bf*: one for transitions to C_1_/C_2_ and another for losses from those states. See Conant (2014) from the main text.

c: *P-*value for the test of the hypothesis that model WGD-*bfc* fits these data no better than WGD-*bf*. Likelihood ratio test with 2 degrees of freedom.

d: log-likelihood for the respective models.

e: Maximum ln-likelihood estimate of the relative duplicate fixation rate for the respective models.

f: Maximum likelihood estimate of the relative rate of retention from the more fractionated genome for the respective models.
