# Supplementary material for: Preferential retention of genes from one parental genome after polyploidy illustrates the nature and scope of the genomic conflicts induced by hybridization
Source: PLoS Genet. 2018 Mar 28;14(3):e1007267. doi: 10.1371/journal.pgen.1007267 (PMC5891031; doi:10.1371/journal.pgen.1007267)
Supplement: S3 Table — (DOCX) [file pgen.1007267.s006.docx]

**S3 Table:** Overrepresented cellular component GO terms

| **GO cellular component ^a^** | **G1 (2549) ^b^** | **G2 (1528) ^c^** | **Duplicate (4162) ^d^** | **G1 to G2 fold ^e^** | **Single to Dupl. fold ^f^** | |
| --- | --- | --- | --- | --- | --- | --- |
| *endoplasmic reticulum part (GO:0044432) ^g^* | *57* | *20* | *83* | *1.71* | *0.95* |  |
| *endoplasmic reticulum (GO:0005783) ^g^* | *117* | *48* | *141* | *1.46* | *1.19* |  |
| mediator complex (GO:0016592) | 8 | 4 | 1 | 1.20 | 12.24** |  |
| plastid membrane (GO:0042170) | 36 | 20 | 21 | 1.08 | 2.72** |  |
| chloroplast membrane (GO:0031969) | 35 | 18 | 20 | 1.17 | 2.71** |  |
| chloroplast part (GO:0044434) | 180 | 94 | 157 | 1.15 | 1.78** |  |
| chloroplast envelope (GO:0009941) | 91 | 52 | 83 | 1.05 | 1.76** |  |
| plastid part (GO:0044435) | 182 | 95 | 161 | 1.15 | 1.76** |  |
| photosynthetic membrane (GO:0034357) | 46 | 21 | 39 | 1.31 | 1.75 |  |
| plastid envelope (GO:0009526) | 92 | 54 | 86 | 1.02 | 1.73** |  |
| thylakoid membrane (GO:0042651) | 45 | 21 | 39 | 1.28 | 1.73 |  |
| thylakoid part (GO:0044436) | 48 | 25 | 44 | 1.15 | 1.69 |  |
| thylakoid (GO:0009579) | 63 | 37 | 61 | 1.02 | 1.67* |  |
| chloroplast stroma (GO:0009570) | 94 | 54 | 92 | 1.04 | 1.64** |  |
| chloroplast thylakoid (GO:0009534) | 53 | 31 | 53 | 1.02 | 1.62 |  |
| plastid stroma (GO:0009532) | 95 | 54 | 95 | 1.05 | 1.60** |  |
| intracellular ribonucleoprotein complex (GO:0030529) | 57 | 31 | 149 | 1.10 | 0.60** |  |
| ribonucleoprotein complex (GO:1990904) | 57 | 31 | 149 | 1.10 | 0.60** |  |
| ribosome (GO:0005840) | 35 | 20 | 104 | 1.05 | 0.54** |  |
| ribosomal subunit (GO:0044391) | 19 | 10 | 71 | 1.14 | 0.42** |  |
| cytosolic part (GO:0044445) | 23 | 11 | 88 | 1.25 | 0.39** |  |
| cytosolic ribosome (GO:0022626) | 22 | 9 | 85 | 1.47 | 0.37** |  |
| large ribosomal subunit (GO:0015934) | 10 | 4 | 48 | 1.50 | 0.30** |  |
| cytosolic large ribosomal subunit (GO:0022625) | 7 | 1 | 45 | 4.20 | 0.18** |  |
| small nucleolar ribonucleoprotein complex (GO:0005732) ^h^ | 2 | 0 | 12 | inf | 0.17 |  |
| proteasome core complex (GO:0005839) ^h^ | 2 | 1 | 13 | 1.20 | 0.24 |  |
| NADH dehydrogenase complex (GO:0030964) ^h^ | 2 | 2 | 16 | 0.60 | 0.26 |  |
| mitochondrial respiratory chain complex I (GO:0005747) ^h^ | 2 | 2 | 16 | 0.60 | 0.26 |  |
| respiratory chain complex I (GO:0045271) ^h^ | 2 | 2 | 16 | 0.60 | 0.26 |  |
| endopeptidase complex (GO:1905369) ^h^ | 3 | 5 | 23 | 0.36 | 0.36 |  |
| proteasome complex (GO:0000502) ^h^ | 3 | 5 | 23 | 0.36 | 0.36 |  |
| respiratory chain complex (GO:0098803) ^h^ | 6 | 3 | 24 | 1.20 | 0.38 |  |
| clathrin-coated vesicle (GO:0030136) ^h^ | 7 | 4 | 29 | 1.05 | 0.39 |  |

** P-value ≤ 0.0001, * P-value ≤ 0.001

a: Analysis type: PANTHER Overrepresentation Test (release 20160715), annotation version and release date: GO Ontology database released 2017-02-28, annotation dataset: GO cellular component complete.

b: Number of single copy genes from parental genome 1.

c: Number of single copy genes from parental genome 2.

d: Number of surviving duplicated genes.

e: G1 fold enrichment relative to G2, >1: overrepresented in G1, <1: overrepresented in G2.

f: Single copy genes (G1 and G2 combined) fold enrichment relative to duplicates (P-value ≤ 0.01, except for the terms noted in g and h), >1: overrepresented in single copy genes, <1: overrepresented in duplicates.

g: These terms are significantly overrepresented in G1 with G2 as reference (*P*-value ≤ 0.05).

h: These terms are significantly overrepresented in duplicates with single copy genes as reference (P-value ≤ 0.01).
