# Supplementary material for: Preferential retention of genes from one parental genome after polyploidy illustrates the nature and scope of the genomic conflicts induced by hybridization
Source: PLoS Genet. 2018 Mar 28;14(3):e1007267. doi: 10.1371/journal.pgen.1007267 (PMC5891031; doi:10.1371/journal.pgen.1007267)
Supplement: S4 Table — (DOCX) [file pgen.1007267.s007.docx]

**S4 Table:** Overrepresented molecular function GO terms

| **GO molecular function ^a^** | **G1 (2549) ^b^** | **G2 (1528) ^c^** | **Duplicate (4162) ^d^** | **G1 to G2 fold ^e^** | **Single to Dupl. fold ^f^** |
| --- | --- | --- | --- | --- | --- |
| *metalloendopeptidase activity (GO:0004222) ^g^* | *11* | *1* | *10* | *6.59** | *1.23* |
| *enzyme inhibitor activity (GO:0004857) ^g^* | *10* | *1* | *22* | *5.99* | *0.51* |
| *solute:cation symporter activity (GO:0015294) ^g^* | *18* | *4* | *18* | *2.70* | *1.25* |
| drug binding (GO:0008144) | 4 | 4 | 1 | 0.60 | 8.17 |
| ATP-dependent DNA helicase activity (GO:0004003) | 6 | 2 | 1 | 1.80 | 8.17 |
| ligase activity, forming aminoacyl-tRNA and related compounds (GO:0016876) ^i^ | 6 | 7 | 2 | 0.51 | 6.64** |
| ligase activity, forming carbon-oxygen bonds (GO:0016875) ^i^ | 6 | 7 | 2 | 0.51 | 6.64** |
| aminoacyl-tRNA ligase activity (GO:0004812) | 6 | 7 | 2 | 0.51 | 6.64** |
| cis-trans isomerase activity (GO:0016859) | 12 | 6 | 3 | 1.20 | 6.13** |
| DNA-dependent ATPase activity (GO:0008094) | 13 | 4 | 3 | 1.95 | 5.78** |
| peptidyl-prolyl cis-trans isomerase activity (GO:0003755) | 12 | 5 | 3 | 1.44 | 5.78** |
| ATP-dependent helicase activity (GO:0008026) | 21 | 9 | 10 | 1.40 | 3.06** |
| purine NTP-dependent helicase activity (GO:0070035) ^i^ | 21 | 9 | 10 | 1.40 | 3.06** |
| helicase activity (GO:0004386) | 28 | 15 | 17 | 1.12 | 2.58** |
| endonuclease activity (GO:0004519) | 26 | 10 | 15 | 1.56 | 2.45* |
| isomerase activity (GO:0016853) | 46 | 25 | 32 | 1.10 | 2.27** |
| ATPase activity, coupled (GO:0042623) | 56 | 27 | 43 | 1.24 | 1.97** |
| structural molecule activity (GO:0005198) ^h^ | 33 | 29 | 106 | 0.68 | 0.60 |
| structural constituent of ribosome (GO:0003735) ^h^ | 26 | 16 | 80 | 0.97 | 0.54 |
| threonine-type endopeptidase activity (GO:0004298) ^h^ | 1 | 1 | 13 | 0.60 | 0.16 |
| threonine-type peptidase activity (GO:0070003) ^h^ | 1 | 1 | 13 | 0.60 | 0.16 |
| clathrin binding (GO:0030276) ^h,i^ | 3 | 0 | 16 | inf | 0.19 |
| transferase activity, transferring acyl groups, acyl groups converted into alkyl on transfer (GO:0046912) ^h,i^ | 1 | 0 | 9 | inf | 0.11 |
| phosphotransferase activity, for other substituted phosphate groups (GO:0016780) ^h,i^ | 0 | 1 | 8 | 0.00 | 0.13 |

** P-value ≤ 0.0001, * P-value ≤ 0.001

a: Analysis type: PANTHER Overrepresentation Test (release 20160715), annotation version and release date: GO Ontology database released 2017-02-28, annotation dataset: GO molecular function complete.

b: Number of single copy genes from parental genome 1.

c: Number of single copy genes from parental genome 2.

d: Number of surviving duplicated genes.

e: G1 fold enrichment relative to G2, >1: overrepresented in G1, <1: overrepresented in G2.

f: Single copy genes (G1 and G2 combined) fold enrichment relative to duplicates (P-value ≤ 0.01, except for the terms noted in g and h), >1: overrepresented in single copy genes, <1: overrepresented in duplicates.

*g:* These terms are significantly overrepresented in G1 with G2 as reference *(P*-value ≤ 0.05)*.*

h: These terms are significantly overrepresented in duplicates with single copy genes as reference (*P*-value ≤ 0.001).

i: Not shown in S3 Fig.
