# Supplementary material for: Preferential retention of genes from one parental genome after polyploidy illustrates the nature and scope of the genomic conflicts induced by hybridization
Source: PLoS Genet. 2018 Mar 28;14(3):e1007267. doi: 10.1371/journal.pgen.1007267 (PMC5891031; doi:10.1371/journal.pgen.1007267)
Supplement: S5 Table — (DOCX) [file pgen.1007267.s008.docx]

**S5 Table:** Overrepresented biological process GO terms

| **GO biological process ^a^** | **G1 (2549)^b^** | **G2 (1528)^b^** | **Duplicate (4162)^d^** | **G1 to G2 fold^e^** | **Single to Dupl. fold^f^** | |
| --- | --- | --- | --- | --- | --- | --- |
| *transcription initiation from RNA polymerase II promoter (GO:0006367) ^g^* | *1* | *6* | *1* | *0.10* | *7.15* | |
| *auxin metabolic process (GO:0009850) ^g^* | *1* | *6* | *14* | *0.10* | *0.51* | |
| phloem or xylem histogenesis (GO:0010087) ^h^ | 13 | 1 | 11 | 7.78** | 1.30 | |
| regulation of multi-organism process (GO:0043900) ^h^ | 13 | 1 | 14 | 7.78** | 1.02 | |
| detoxification (GO:0098754) ^h^ | 9 | 1 | 5 | 5.39 | 2.04 | |
| cellular polysaccharide biosynthetic process (GO:0033692) ^h^ | 28 | 7 | 29 | 2.40 | 1.23 | |
| protein folding (GO:0006457) ^h^ | 37 | 11 | 30 | 2.02 | 1.63 | |
| cellular component organization or biogenesis (GO:0071840) ^h^ | 243 | 183 | 401 | 0.80 | 1.08 | |
| cellular component biogenesis (GO:0044085) ^h^ | 81 | 73 | 157 | 0.67 | 1.00 | |
| cell cycle (GO:0007049) ^h^ | 37 | 41 | 49 | 0.54 | 1.63 | |
| ribonucleoprotein complex biogenesis (GO:0022613) ^h^ | 21 | 28 | 55 | 0.45 | 0.91 | |
| rhythmic process (GO:0048511) ^h^ | 7 | 15 | 23 | 0.28 | 0.98 | |
| circadian rhythm (GO:0007623) ^h^ | 6 | 13 | 19 | 0.28 | 1.02 | |
| cell cycle process (GO:0022402) ^h^ | 29 | 36 | 32 | 0.48 | 2.07** | |
| DNA-templated transcription, initiation (GO:0006352) ^h^ | 2 | 10 | 1 | 0.12 | 12.24** | |
| reciprocal DNA recombination (GO:0035825) ^j^ | 6 | 6 | 1 | 0.60 | 12.24** | |
| reciprocal meiotic recombination (GO:0007131) | 6 | 6 | 1 | 0.60 | 12.24** | |
| tRNA aminoacylation for protein translation (GO:0006418) | 5 | 6 | 1 | 0.50 | 11.22** | |
| monocarboxylic acid transport (GO:0015718) | 6 | 5 | 1 | 0.72 | 11.22** | |
| protein peptidyl-prolyl isomerization (GO:0000413) | 9 | 2 | 1 | 2.70 | 11.22** | |
| tRNA aminoacylation (GO:0043039) ^j^ | 5 | 6 | 1 | 0.50 | 11.22** | |
| amino acid activation (GO:0043038) ^j^ | 5 | 6 | 1 | 0.50 | 11.22** | |
| ketone biosynthetic process (GO:0042181) | 4 | 6 | 1 | 0.40 | 10.20** | |
| defense response to fungus, incompatible interaction (GO:0009817) | 6 | 3 | 1 | 1.20 | 9.18* | |
| nuclear chromosome segregation (GO:0098813) | 7 | 10 | 2 | 0.42 | 8.67** | |
| regulation of cellular amino acid metabolic process (GO:0006521) | 5 | 3 | 1 | 1.00 | 8.16 | |
| regulation of cellular amine metabolic process (GO:0033238) ^j^ | 5 | 3 | 1 | 1.00 | 8.16 | |
| positive regulation of protein complex assembly (GO:0031334) | 4 | 4 | 1 | 0.60 | 8.16 | |
| RNA secondary structure unwinding (GO:0010501) | 4 | 4 | 1 | 0.60 | 8.16 | |
| cellular ketone metabolic process (GO:0042180) | 6 | 8 | 2 | 0.45 | 7.14** | |
| meiosis I (GO:0007127) | 8 | 6 | 2 | 0.80 | 7.14** | |
| regulation of cell differentiation (GO:0045595) | 9 | 4 | 2 | 1.35 | 6.63* | |
| nucleobase metabolic process (GO:0009112) | 10 | 2 | 2 | 3.00 | 6.12* | |
| transcription from RNA polymerase II promoter (GO:0006366) | 5 | 10 | 3 | 0.30 | 5.10* | |
| meiotic nuclear division (GO:0007126) | 9 | 11 | 4 | 0.49 | 5.10** | |
| recombinational repair (GO:0000725) ^j^ | 9 | 5 | 3 | 1.08 | 4.76 | |
| double-strand break repair via homologous recombination (GO:0000724) | 9 | 5 | 3 | 1.08 | 4.76 | |
| starch metabolic process (GO:0005982) | 16 | 6 | 5 | 1.60 | 4.49** |  |
| chromosome segregation (GO:0007059) | 8 | 11 | 5 | 0.44 | 3.88* |  |
| double-strand break repair (GO:0006302) | 13 | 5 | 5 | 1.56 | 3.67 |  |
| cellular aldehyde metabolic process (GO:0006081) | 17 | 8 | 7 | 1.27 | 3.64** |  |
| meiotic cell cycle process (GO:1903046) | 11 | 13 | 8 | 0.51 | 3.06 |  |

**S5 Table continued**

| **GO biological process ^a^** | **G1 (2549) ^b^** | **G2 (1528) ^c^** | **Duplicate (4162) ^d^** | **G1 to G2 fold ^e^** | **Single to Dupl. fold ^f^** |
| --- | --- | --- | --- | --- | --- |
| tRNA metabolic process (GO:0006399) | 12 | 11 | 8 | 0.65 | 2.93 |
| DNA recombination (GO:0006310) | 15 | 10 | 9 | 0.90 | 2.83 |
| cellular response to radiation (GO:0071478) | 15 | 14 | 11 | 0.64 | 2.69 |
| nuclear division (GO:0000280) | 20 | 20 | 16 | 0.60 | 2.55* |
| chloroplast organization (GO:0009658) | 18 | 13 | 13 | 0.83 | 2.44 |
| ncRNA metabolic process (GO:0034660) | 21 | 26 | 20 | 0.48 | 2.40** |
| plastid organization (GO:0009657) | 23 | 16 | 18 | 0.86 | 2.21 |
| organelle fission (GO:0048285) | 25 | 23 | 24 | 0.65 | 2.04 |
| transmembrane transport (GO:0055085) | 67 | 40 | 64 | 1.00 | 1.71* |
| intracellular signal transduction (GO:0035556) | 50 | 31 | 129 | 0.97 | 0.64 |
| protein catabolic process (GO:0030163) | 34 | 19 | 100 | 1.07 | 0.54* |
| cellular protein catabolic process (GO:0044257) | 31 | 19 | 97 | 0.98 | 0.53* |
| ubiquitin-dependent protein catabolic process (GO:0006511) | 27 | 19 | 90 | 0.85 | 0.52* |
| modification-dependent macromolecule catabolic process (GO:0043632) ^j^ | 27 | 19 | 90 | 0.85 | 0.52* |
| modification-dependent protein catabolic process (GO:0019941) ^j^ | 27 | 19 | 90 | 0.85 | 0.52* |
| response to ethylene (GO:0009723) | 24 | 13 | 74 | 1.11 | 0.51 |
| proteolysis involved in cellular protein catabolic process (GO:0051603) | 29 | 19 | 97 | 0.91 | 0.51** |
| clathrin coat assembly (GO:0048268) ^j^ | 0 | 0 | 12 | NA | 0.00 |
| response to freezing (GO:0050826) ^i,j^ | 1 | 0 | 12 | inf | 0.09 |
| positive regulation of protein kinase activity (GO:0045860) ^i,j^ | 1 | 0 | 10 | inf | 0.10 |
| positive regulation of kinase activity (GO:0033674) ^i,j^ | 1 | 0 | 10 | inf | 0.10 |
| positive regulation of phosphorylation (GO:0042327) ^i,j^ | 1 | 0 | 10 | inf | 0.10 |
| positive regulation of protein phosphorylation (GO:0001934) ^i,j^ | 1 | 0 | 10 | inf | 0.10 |
| positive regulation of phosphorus metabolic process (GO:0010562) ^i,j^ | 1 | 0 | 10 | inf | 0.10 |
| positive regulation of phosphate metabolic process (GO:0045937) ^i,j^ | 1 | 0 | 10 | inf | 0.10 |
| regulation of protein phosphorylation (GO:0001932) ^i,j^ | 2 | 0 | 15 | inf | 0.14 |
| regulation of protein kinase activity (GO:0045859) ^i,j^ | 2 | 0 | 13 | inf | 0.16 |
| regulation of phosphorylation (GO:0042325) ^i,j^ | 3 | 0 | 17 | inf | 0.18 |
| regulation of phosphorus metabolic process (GO:0051174) ^i^ | 6 | 2 | 24 | 1.80 | 0.34 |
| response to gibberellin (GO:0009739) ^i^ | 9 | 7 | 39 | 0.77 | 0.42 |

** P-value ≤ 0.0001, * P-value ≤ 0.001

a: Analysis type: PANTHER Overrepresentation Test (release 20160715), annotation version and release date: GO Ontology database released 2017-02-28, annotation dataset: GO biological process complete.

b: Number of single copy genes from parental genome 1.

c: Number of single copy genes from parental genome 2.

d: Number of surviving duplicated genes.

e: G1 fold enrichment relative to G2, >1: overrepresented in G1, <1: overrepresented in G2.

f: Single copy genes (G1 and G2 combined) fold enrichment relative to duplicates (*P*-value ≤ 0.01, except for the terms noted in g, h and i), >1: overrepresented in single copy genes, <1: overrepresented in duplicates.

g: These terms are significantly overrepresented in G2 with G1 as reference *(P*-value ≤ 0.05).

h: These terms are significantly overrepresented in G1 with G2 as reference (P-value ≤ 0.05).

i: These terms are significantly overrepresented in duplicates with single copy genes as reference (P-value ≤ 0.01).

j: Not shown in S3 Fig.
